# Glass Female Syringes

**Published:** 1856-01

**Authors:** Frank H. Hamilton


ART. III.— *Glass Female Syringes.* By FRANK H. HAMILTON, M. D.

Gradually the old pewter female syringes have gone into disuse, and physicians have substituted the neater, and perhaps, cheaper glass syringes; but the occurrence of several accidents, in some of which my surgical services have been required, induce me to question the value of the substitution.

In three instances I have been called upon to remove from the vagina the broken fragments of these syringes. In one instance the accident occurred in consequence of the sudden alarm of the female while she was using the syringe, and was in no measure, probably, due to the imperfection of the instrument; but in the two other cases the glass gave way from the mere pressure of the fluid while the piston was ascending—the accident, certainly, might occur when the piston fits snugly even though there was no defect in the glass: but if there chances to be the slightest irregularity in the walls of the syringe, or a fissure, such as might easily escape detection, or an unusual tenuity of the round extremity, a fracture would be almost certain to follow. It was this latter circumstance, viz., the extreme thinness of the extremity of the instrument, which occasioned the accident in two of the cases mentioned. Two other cases have been related to me as having occurred in the practice of neighboring physicians, making in all five that have come to my knowledge.

It is probable that no examination, however critical, would enable us to determine, before the fracture has taken place, whether the end of the instrument has a suitable thickness, and it is very likely that a majority of them are blown too thin for safety. I have found the fragments not as thick as my finger nail.

Of late I have, therefore, uniformly recommended either some appropriate metallic instrument, or perhaps more generally the gum elastic bag with an ivory nozzle.

